# Efficacy of Low-Carbohydrate Ketogenic Diet as an Adjuvant Cancer Therapy: A Systematic Review and Meta-Analysis of Randomized Controlled Trials

**DOI:** 10.3390/nu13051388

**Published:** 2021-04-21

**Authors:** Ya-Feng Yang, Preety Babychen Mattamel, Tanya Joseph, Jian Huang, Qian Chen, Babatunde O. Akinwunmi, Casper J. P. Zhang, Wai-Kit Ming

**Affiliations:** 1 School of Medicine, Jinan University, Guangzhou 510632, China; mapleyang@um.edu.my (Y.-F.Y.); pret22@stu2014.jnu.edu.cn (P.B.M.); tanyajoseph1996@yahoo.com (T.J.); chenqian@stu2020.jnu.edu.cn (Q.C.); 2 International School, Jinan University, Guangzhou 510632, China; 3 Faculty of Medicine, University of Malaya, Kuala Lumpur 50603, Malaysia; 4 Department of Epidemiology and Biostatistics, School of Public Health, St Mary’s Campus, Imperial College London, London W2 1PG, UK; jian.huang@imperial.ac.uk; 5 Brigham and Women’s Hospital, Harvard Medical School, Boston, MA 02115, USA; bakinwunmi@bwh.harvard.edu; 6 School of Public Health, LKS Faculty of Medicine, University of Hong Kong, Hong Kong, China; casperz1@connect.hku.hk

**Keywords:** low-carbohydrate diet, ketogenic diet, randomized controlled trials, cancer, adjuvant cancer therapy

## Abstract

Background: The role of low-carbohydrate ketogenic diet (LCKD) as an adjuvant therapy in antitumor treatment is not well established. This systematic review and meta-analysis of randomized controlled trials (RCTs) was conducted to investigate the efficacy of LCKD as an adjuvant therapy in antitumor treatment compared to non-ketogenic diet in terms of lipid profile, body weight, fasting glucose level, insulin, and adverse effects; Methods: In this study, databases such as PubMed, Web of Science, Scopus, CINAHL, and Cochrane trials were searched. Only RCTs that involved cancer participants that were assigned to dietary interventions including a LCKD group and a control group (any non-ketogenic dietary intervention) were selected. Three reviewers independently extracted the data, and the meta-analysis was performed using a fixed effects model or random effects model depending on the I^2^ value or *p*-value; Results: A total of six articles met the inclusion/exclusion criteria. In the overall analysis, the post-intervention results = standard mean difference, SMD (95% CI) showed total cholesterol (TC) level = 0.25 (−0.17, 0.67), HDL-cholesterol = −0.07 (−0.50, 0.35), LDL-cholesterol = 0.21 (−0.21, 0.63), triglyceride (TG) = 0.09 (−0.33, 0.51), body weight (BW) = −0.34 (−1.33, 0.65), fasting blood glucose (FBG) = −0.40 (−1.23, 0.42) and insulin = 0.11 (−1.33, 1.55). There were three outcomes showing significant results in those in LCKD group: the tumor marker PSA, *p* = 0.03, the achievement of ketosis *p* = 0.010, and the level of satisfaction, *p* = 0.005; Conclusions: There was inadequate evidence to support the beneficial effects of LCKDs on antitumor therapy. More trials comparing LCKD and non-KD with a larger sample size are necessary to give a more conclusive result.

## 1. Introduction

Low-carbohydrate ketogenic diet (LCKD) is defined as the daily consumption of fewer than 50 g (around 10% of daily energy intake) of carbohydrates, regardless of fat, protein, or caloric intake [1]. Clinically used KDs mainly have a fat to carbohydrate and protein ratio of at least 2:1 to 3:1, meaning that the percentage of calories from fat is a minimum of 80%. For LCKDs, fat usually makes up more than 80% and protein about 10% of daily energy intake [2]. In the most recent decades, LCKDs have been promoted for weight loss and diabetes, but the effectiveness of LCKDs has remained uncertain. In addition, LCKDs have shown therapeutic uses in multiple neurological disorders including epilepsy, Alzheimer’s and Parkinson’s disease, headaches, sleep disorder, autism, brain cancer, and multiple sclerosis [3]. LCKDs were used to control glycemic level in diabetes type 2, obesity, hypercholesterolemia, and polycystic ovary syndrome [4,5].

LCKDs have recently been proposed as an adjuvant therapy in anticancer treatment [6]. LCKDs are fasting mimicking diets that cause increase in ketone bodies without restricting the energy intake [7]. This concept of using LCKDs as adjuvant therapy for cancer is based on the mechanism of the Warburg effect [8]. The Warburg effect is the process where cancer cells mainly derive their energy source of ATP through glycolysis instead of oxidative phosphorylation, which causes some cancer cells to lose the capability to metabolize ketone bodies. This leads to the reasoning behind the theory that LCKDs may be beneficial to patients because a reduction in glucose can cause the cancer cells being unable to derive their energy through glycolysis and, therefore, ketosis will take place and the normal cells will adapt to utilizing ketone bodies. This will also cause a reduction in insulin and insulin-like growth factors, which are known essential factors for the proliferation of cancer cells [9]. By creating such an uncomfortable environment for cancer cells, the use of LCKDs may have a beneficial effect towards treatment therapy for cancer alongside surgery, chemotherapy, radiotherapy, or immunotherapy by enhancing antitumor effects and could have an overall improvement in the quality of life of the patients [2]. This can be a significant boost in the medical field when treating cancer [10].

The effectiveness of LCKDs as an adjuvant therapy in antitumor treatment has been debated over the years. Generally, this meta-analysis is conducted through obtaining results from the limited randomized controlled trials (RCTs) based on LCKDs as adjuvant therapy on cancer patients and analyzing the results for the significance towards lipid profile, body weight, fasting blood glucose, insulin, tumor markers, ketosis, adverse events, and satisfaction levels of the patients. We aim to assess the effectiveness of LCKDs compared to non-ketogenic diets as an adjuvant therapy in cancer patients undergoing surgery, chemotherapy, radiotherapy, immunotherapy, or post-operative recovery. We also aim to investigate the side effects that came along with the LCKD diet to provide more information about the outcome(s) in anticancer treatment of LCKDs.

## 2. Materials and Methods

This review was conducted according to the standard procedure developed by the Cochrane Collaboration in which the studies were included employing the PICOS (Participants, Intervention, Comparison and Outcome and Study design) principle. (See Table 1).

### 2.1. Search Strategy

The literature search was conducted from 21 to 30 December 2019 by two authors (Y.F. and P.M.). A comprehensive search was performed in PubMed, Web of Science, Scopus, CINAHL, and Cochrane trials following the Preferred Reporting Items for Systematic reviews and Meta-Analysis (PRISMA; the checklist is available in Table S1) [11]. Searches were limited to studies conducted on humans from each database’s inception until 20 December 2019 using the following combinations of search terms: (“KD *” OR ((“Ketogenic *” OR “Keto *” OR “Low carb *” OR “Low-carb *” OR “High fat *” OR “High-fat *” OR “medium-chain triglyceride *” OR “medium-chain triglyceride *” OR “MCT *” OR “Atkin *”) AND (“Diet *” OR “Plan *” OR “Treat *”))) AND (“Neoplasm *” OR “Cancer *” OR “Tumo *” OR “Carcinoma *” OR “Malignan *” OR “Ongolog *” OR “Metastas *” OR “Lymphoma *” OR “leukemia” OR “Adenoma *” OR “Adenocarcinoma *” OR “Glioma *” OR “Sarcoma *”) AND (“Randomized controlled trial *” OR “Controlled clinical trial *” OR “Random *” OR “RCT*”) (for details, please see Table 2). A ketogenic diet (KD) was defined as any dietary manipulation of high fats, moderate proteins, and very low carbohydrates [12]. Studies that did not have KD as the intervention were excluded. In addition, two additional studies [13,14] were obtained by YF and PM through manual searching from the reference lists of relevant literature on Google Scholar and other databases during record screening.

### 2.2. Inclusion and Exclusion Criteria

The inclusion criteria were designed according to the PICOS principle (see Table 1). Only randomized controlled trials that met the following criteria were selected: [1] The study participants were patients diagnosed with cancer/tumor. [2] Dietary intervention must include KD (or the subtype of KD) and a control group (any dietary intervention); [3] Written in English; [4] Basic information required for meta-analysis such as demographic characteristics of the subjects, number of enrolled patients, number of adherent and dropouts, duration of the trials and [5] The preceding 4 main points were included without limitations to geographical region, race and age. Articles were excluded if they [1] Were non-randomized; [2] Have no comparison group; [3] Were non-human species; [4] Were conference abstracts, book chapters, reviews, or other forms without detailed empirical data and [5] Have no exposure or outcome of interest.

Based on the above inclusion and exclusion criteria, the titles and abstracts of the selected articles were screened independently by three authors who were not blinded to the authors and the article titles. The full-text versions of potentially eligible articles were retrieved for further evaluation. Any discrepancy that occurred during this process was resolved by consensus.

### 2.3. Data Extraction and Quality Assessment

The two authors (Y.F., P.M.) extracted the relevant data independently using a Microsoft Excel customized sheet for a data extraction based on the PICOS principle. Any discrepancy was settled through joint discussion with the third author (TJ). The corresponding author was contacted through email for missing data.

The following information was extracted: first author, publication year, study design, age of the participants, intervention measures, cancer type, study size, number of cases, duration of follow-up, and outcome (body weight, lipid profile, biochemical indices, tumor markers level etc.). When the studies measured outcomes in a variety of ways, the result were converted to a uniform scale.

The primary outcome sought in the studies was the post-intervention result of the lipid profile (cholesterol, HDL-cholesterol, LDL-cholesterol, triglycerides in mg/dL), fasting blood glucose in mg/dL, insulin in µU/mL (pg/mL was converted to µU/mL by using the Formula ÷1000 ng/mL÷0.04 ) [15], CEA in ng/mL, CA19-9 in U/mL, PSA in ng/mL, TNF-alpha in pg/mL and weight in kg. To extract numerical data published as figures in the articles, we used Web Plot Digitizer 4.2, available online [16]. The information was extracted from the published articles, protocols, and commentaries related to each study.

Study quality was assessed according to the Cochrane Handbook recommendations using the “risk of bias” method. The method classifies bias in randomized studies as “low”, “high”, or “unclear” on the basis of the presence or absence of seven processes (random sequence generation, allocation concealment, blinding participants and personnel, blinding of outcome assessment, incomplete outcome data, selective reporting, and other bias) [17,18].

### 2.4. Statistical Analysis

Review Manager software (Version 5.3) was utilized for statistical analysis. We used Cochran–Mantel–Haenszel test (CMH) and inverse-variance method to perform a meta-analysis. Continuous variables of *N*, mean, standard deviation, and median (25th percentile, 75th percentile) were extracted from each intervention and control group of the included studies. All the resulting variables were uniformly converted to mean ± standard deviation (SD) for merging. For the original study that reported only the median, we converted the median of baseline and post-intervention data to mean ± SD by calculating the closest approximation of mean and SD from the median and interquartile range (IQR) [19,20,21,22]. To do this, standardized mean differences (SMD) and their 95% confidence intervals (CI) were calculated to assess the change in each selected variable. During the analysis process, all the standard errors of the mean (SEM) were converted into SD by using the formula SD=SEM×√N [17]. The Q test and the I^2^ test were used to evaluate the heterogeneity of similar studies. I^2^ value > 50% or *p*-value < 0.10 was statistically significant and the random-effects model would be selected. Otherwise, the fixed effects model would be used. If significant heterogeneity was exhibited, the subgroup analysis was performed to explore the potential source of heterogeneity. For outcomes that were unable to be combined across trials, a narrative synthesis was presented.

## 3. Results

### 3.1. Study Selection

**Figure 1:**
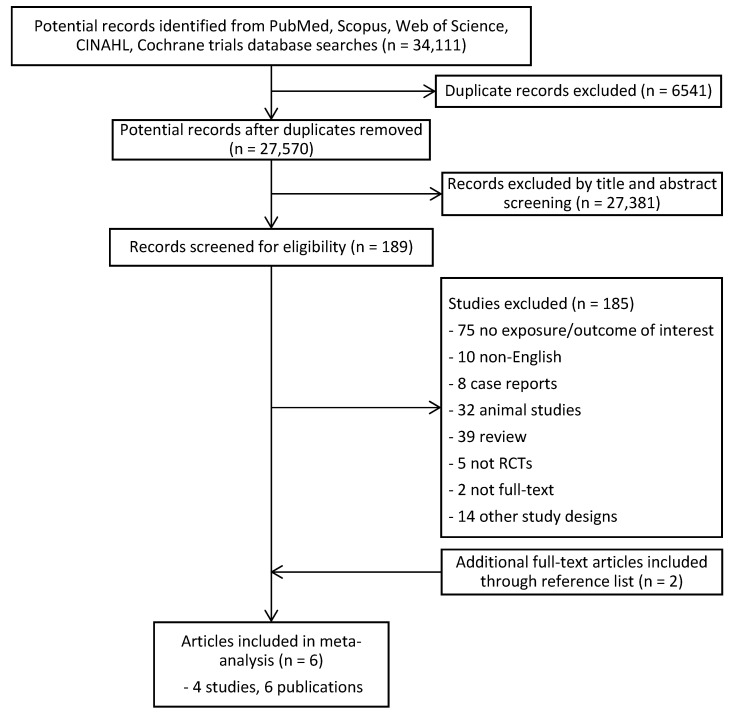
Flow diagram of the literature search process.

A total of 34,111 articles were identified. After the removal of 6541 duplicate records, 27,570 potential records were left. A total of 27,381 articles were excluded based on their titles and abstracts, resulting in 189 full-text articles being assessed for eligibility, as shown in Figure 1. Flow diagram of the literature search process. From that, 185 were excluded. In addition, two studies [13,14] were obtained through manual searching. Finally, a total of six studies were included for meta-analysis. Among the six published articles, two of the articles [13] and [23] were published based on the same cohort subjects from articles [14] and [24], respectively.

### 3.2. Study Characteristics

Table 3 shows the general characteristics of included studies. The trials included a total of 222 individuals (excluding data from articles [13,23] as these articles shared the same data with articles [14,24] respectively) in which 153 individuals had completed the trials (79 on low-carbohydrate diets and 74 on general diet or American cancer society diet), and the mean duration of the trials varied from 4 to 24 weeks. Table 4 shows the summary of the patient data from baseline comparing the intervention group and control group.

Among the six articles, five of them [13,14,23,24,25] showed data on lipid profile (cholesterol, HDL-cholesterol, LDL-cholesterol, and triglyceride), two were from the same study but reported different numbers of participants; one of the studies [26] exhibited data as a linear graph and, thus, it was not possible to extract the data from it.

### 3.3. Study Quality of Trials

**Figure 2:**
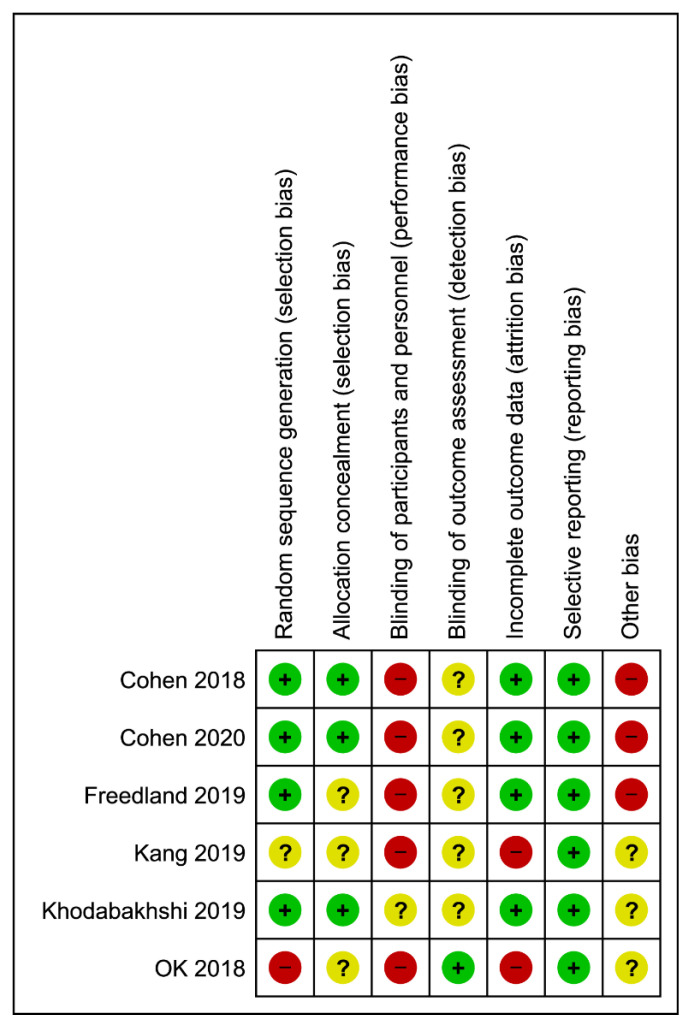
Assessment of risk of bias of the studies in the meta-analysis.

Results from the quality assessment are provided in Figure 2. Most studies showed low risk for random sequence generation by using either computer-generated blocked randomization, block balanced randomization, or permitted block design randomization. One study showed high risk of bias [14] while one did not report specific information of the random sequence generation [13]. Half of the studies showed unclear risk for allocation concealment of the randomization due to insufficient information provided regarding sequence generation process [13,14,25]. Blinding of participants was impossible for most of the studies due to the nature of trials, it was not possible to blind dietary intakes in a free-living environment. Among the included studies, no clear information reported on the blinding of outcome assessment during the intervention or analysis stage except in one study [14]. Two studies showed high risk in attrition bias due to significantly uneven dropout of participants during follow-up, in which the losses were likely to influence the final outcome [13,14]. None of the trials underwent selective reporting bias. Detailed judgement for the risk of bias was available in Table S2.

### 3.4. Findings

#### 3.4.1. Lipid Profile

##### Total Cholesterol (TC)

Among all six included articles, three articles were assessed for lipid profile, three of which show the results from baseline and post intervention. The pooled analysis using a fixed effects model at baseline for total cholesterol (TC) level [SMD (95% CI) = −0.20 (−0.62, 0.23), I^2^ = 56% indicates moderate heterogeneity. The effect of the baseline subgroup was *p* = 0.36, which was not significant. Meanwhile, for post-intervention, it was identified that the non-ketogenic diet (non-KD) group had higher TC level [SMD (95% CI) = 0.25 (−0.17, 0.67), and the heterogeneity in this subgroup did not prove to be significant, I^2^ = 0%. As overall effect *p* = 0.24, there was also no significant effect in this subgroup. The test for subgroup differences suggested that there was no proven significant subgroup effect (*p* = 0.14) and moderate heterogeneity between the subgroups since I^2^ = 53.4%.

##### HDL-Cholesterol

Three articles reported results on HDL cholesterol. Fixed effect model was used in this pool analysis, and there was no significant subgroup effect for the studies of HDL-cholesterol at baseline (*p* = 0.24). Similarly, for the post-intervention subgroup of HDL-cholesterol, the value for [SMD (95% CI) = −0.07 (−0.50, 0.35) indicated very small effect size of HDL-cholesterol in KD group, where the increase of HDL-cholesterol favored KD group over non-KD group. However, the overall effect for this subgroup was insignificant (*p* = 0.74) and under substantial heterogeneity (I^2^ = 69%). In addition to that, there was low heterogeneity between the subgroup differences, I^2^ = 9.9%, and the subgroup effect was also insignificant (*p* = 0.29).

##### LDL-Cholesterol

In the subgroup analysis of three studies that included LDL results, LDL-cholesterol showed baseline subgroup [SMD (95% CI) = −0.20 (−0.62,0.23), I^2^ = 61% (moderate heterogeneity) while post-intervention subgroup [SMD (95% CI) = 0.21 (−0.21,0.63), I^2^ = 0%. There was no significant heterogeneity in the post-intervention subgroup, and it showed that the individuals assigned to the non-KD group had a small to moderate effect size, the LDL-cholesterol was lower in the non-KD group, meaning the effect of LDL-cholesterol was most likely to favor the non-KD group in the post-intervention subgroup. The test for subgroup differences show *p* = 0.18, I^2^ = 44.5% suggesting that there was no significant effect between baseline and post-intervention subgroups to the intervention of KD and non-KD groups but there was moderate heterogeneity between the subgroups.

##### Triglyceride (TG)

**Figure 3:**
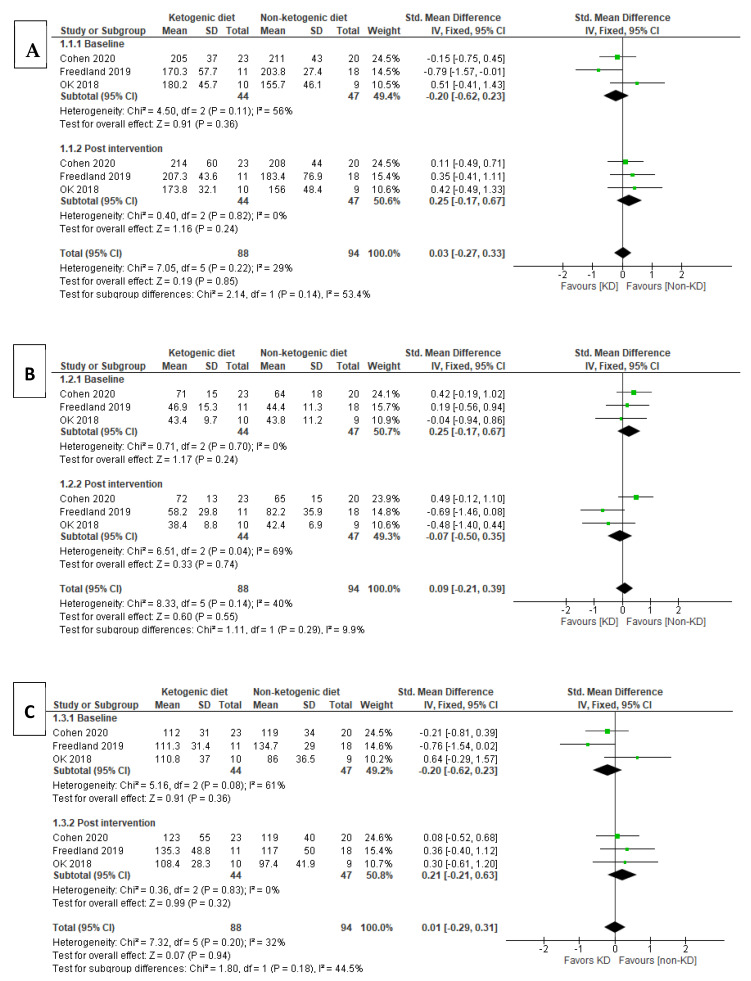

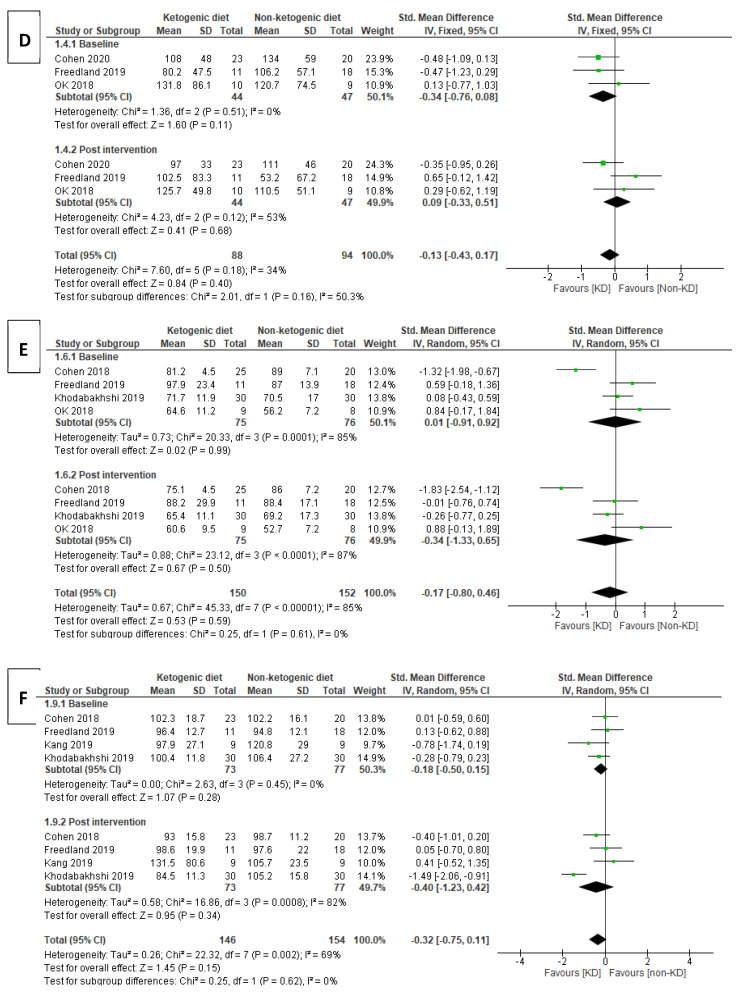

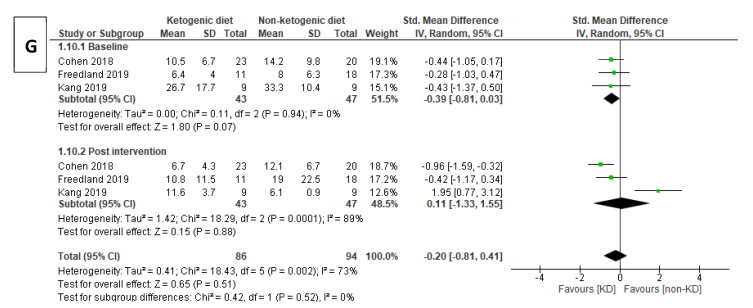
Forest plot for the subgroup effects of the baseline versus post-intervention of ketogenic diet (KD) vs. non-ketogenic diet on TC (**A**), HDL-c (**B**), LDL-c (**C**), TG (**D**), body weight (**E**), fasting blood glucose (**F**), and insulin (**G**).

Three studies were analyzed for triglyceride (TG) levels. For subgroup analysis from Figure 3D, the test for subgroup differences indicated that there was no statistically significant effect (*p* = 0.16), suggesting that TG levels in pre- and post-interventions did not modify the effect of KD in comparison to non-KD groups. The pooled analysis used a fixed effect model to test for TG showed baseline subgroup [SMD (95% CI) = −0.34 (−0.76, 0.08) and [SMD (95% CI) = 0.09 (−0.33, 0.51) for the post-intervention subgroup. The KD group was favored over non-KD group for baseline study while the non-KD group was favored over the KD group for the post-intervention subgroup and, therefore, the subgroup effect is qualitative. Subgroup differences showed I^2^ = 50.3% and indicated that the results from all trials included in this analysis were of moderate heterogeneity. Subgroup differences showed no significant effect between the subgroups of the interventions (*p* = 0.16).

#### 3.4.2. Body Weight (BW)

A subgroup analysis of four studies involving weight was performed. In the analysis for BW, the post-intervention subgroup showed SMD [(95% CI) = −0.34 (−1.33, 0.65), and the KD group has a small–moderate effect size on BW in this subgroup. As seen in Figure 3E, the overall effect for studies in baseline was *p* = 0.99 while in post-intervention was *p* = 0.50. The overall effects of both subgroups were insignificant. Both subgroups showed high levels of heterogeneity (baseline: I^2^ = 85%, post-intervention: I^2^ = 87%). There was no statistically significant subgroup effect (*p* = 0.61).

#### 3.4.3. Fasting Blood Glucose (FBG)

Four studies were analyzed for FBG level. The baseline value of FBG in KD and non-KD groups showed [SMD (95% CI) = −0.18 (−0.50, 0.15), the I^2^ = 0% (homogeneity). Post-intervention [SMD (95% CI) = −0.40 (−1.23, 0.42) where the standardized mean value was lower in the KD group. The FBG level has moderate effect size on the KD group compared to the non-KD group. The FBG level showed high heterogeneity between the studies in the post-intervention subgroup: I^2^ = 82%. The overall subgroup effects showed no statistical significance (*p* = 0.62), I^2^ = 0% (homogeneity).

#### 3.4.4. Insulin

In the analysis of the insulin level in the three studies, 43 participants were in the KD group while forty-seven participants in the non-KD group. I^2^ was 0% at the baseline subgroup. I^2^ = 89% in post-intervention subgroup and [SMD (95% CI) = 0.11 (−1.33, 1.55) for KD versus non-KD groups. The insulin level in the post-intervention group showed a favorable effect on the non-KD group as the standardized (std) mean value was lower but no significant difference between the KD and non-KD groups (*p* = 0.88). There was no heterogeneity between the two subgroups, I^2^ = 0%.

#### 3.4.5. Tumor Marker Levels (CEA, CA19-9) and Prostate-Specific Antigen (PSA) Test

The study reported on tumor markers CEA and CA19-9. The duration from baseline to post-intervention was 2 weeks. A fixed effect model was used to analyze the study. The baseline for CEA showed [SMD (95% CI) = −0.18(−1.10, 0.75) while the SMD (95% CI) for post-intervention was −0.44 (−1.28, 0.50). Both std mean values of CEA showed negative effects, which suggested that the std mean value was lower in KD group. The *p*-value = 0.69, the subgroup effect was not statistically significant. The heterogeneity between the subgroups was not proven significant as I^2^ = 0%. Results for CA19-9 showed baseline [SMD (95% CI) = −0.52 (−1.46, 0.42) and post-intervention [SMD (95% CI) = −0.77 (−1.74, 0.19), and the negative mean values suggested the reduced in CA19-9 favors KD group compared to non-KD group. The subgroup effect was not significant, *p* = 0.71. There was no heterogeneity between the subgroups, I^2^ = 0%.

The study has reported PSA random effects model at baseline [SMD (95% CI) = −0.63 (−1.40, 0.14), post-intervention [SMD (95% CI) = 0.56 (−0.21, 1.33). The mean value of PSA in the post-intervention subgroup indicated a more favorable effect in the non-KD group than KD group. The test for subgroup differences suggested that there was a statistically significant subgroup effect (*p* = 0.03). However, there was a significant heterogeneity between the subgroups, I^2^ = 78.3%.

#### 3.4.6. Ketosis

**Figure 4:**
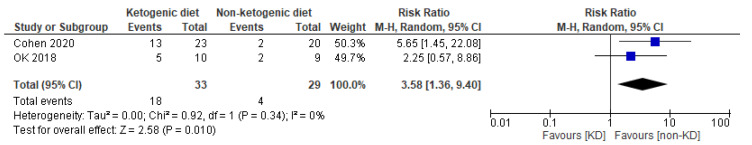
Forest plot of risk ratio in random effects model for ketosis in comparison of KD and non-KD group.

Studies that reported ketosis (see Figure 4) have shown that the KD group has a higher chance of ketosis than the non-KD group, risk ratio (RR) = 3.58, 95% CI = 1.36, 9.40. Homogeneity is achieved, I^2^ = 0%. There is a statistically significant difference between the groups (*p* = 0.010).

#### 3.4.7. Adverse Event

**Figure 5:**
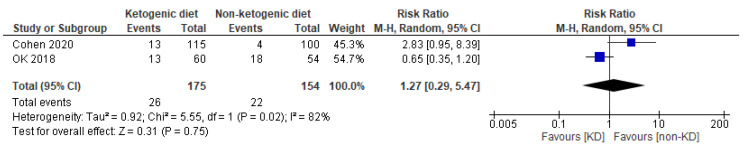
Forest plot of risk ratio in random effects model for adverse events in comparison of KD and non-KD group.

There were three articles that reported adverse effects of the intervention (See Figure 5). However, the data [25] were not extracted as the result was reported by occurrence frequency instead of the number of adverse effects that occurred among the patients. In this study, participants who underwent KD had increased likelihood of getting adverse effects, risk ratio (RR) = 1.27, 95% CI = 0.29, 5.47. The heterogeneity is statistically significant, I^2^ = 82%. There was no strong evidence that KD has an effect, causing an adverse event (*p* = 0.75).

#### 3.4.8. Satisfaction

**Figure 6:**
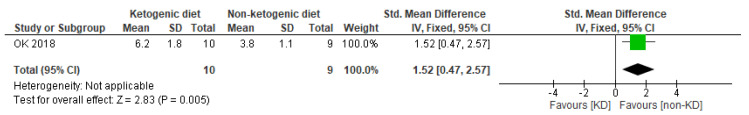
Forest plot of standardized mean difference in fixed effects model for ketosis in comparison of KD and non-KD group.

The fixed effects model in Figure 6 showed satisfaction [SMD (95% CI) = 1.52 (0.47, 2.57) in favor of the non-KD group. The *p*-value = 0.005 which indicated statistically significant differences in the effects on satisfaction between KD and non-KD interventions.

## 4. Discussion

A ketogenic diet is a process that simulates hunger and forces fat to fuel the body by limiting carbohydrate supply. When fat is broken down in the body, it produces ketones; hence, a diet that uses fat as its main energy source to continuously produce ketones is called a ketogenic diet. The principle of the ketogenic diet is related to the energy utilization mechanism. There are three sources of energy in the human body: carbohydrates, fats, and proteins. Some carbohydrates are broken down into glucose, which provides energy, and some are stored in the liver, where they form glycogen. When the body needs energy, it first uses glucose, then liver glycogen; if neither of those stocks is enough, it starts to use fat as a source of energy.

Many studies have shown that cancer cells break down glucose to produce lactic acid, rather than carbon dioxide and water, even in an aerobic environment. Cancer cells tend to use glycolysis instead of the aerobic cycle as in normal cells. This phenomenon, in which glycolysis is mainly used instead of the normal cells’ aerobic cycle, is called the Warburg effect [8]. Increased glycolysis, reduced tricarboxylic acid cycle activity, and oxidative phosphorylation is found early in tumorigenesis, characteristic of tumors [27]. This means that tumor growth is highly dependent on glucose and glycolysis. The ketogenic diet, which simulates a fasting state, mainly relies on fat for energy and reduces the concentration of glucose in the body, which may form an environment that is not conducive to the growth of tumor cells, thus achieving the purpose of inhibiting tumor growth.

In addition, ketogenic diet enhances the oxidative stress response in tumor cells through glucose metabolism and lipid metabolism, respectively. On the one hand, a high-fat, low-carbohydrate diet reduces the ability of tumor cells to synthesize NADPH via the pentose-phosphate pathway, and on the other hand, the oxidative decomposition of fatty acids must go through the mitochondrial oxidative phosphorylation pathway. However, due to damage or defect of the electron transport chain in the mitochondria of tumor cells, more electrons will leak out, making it easier for superoxide anions to acquire electrons and generate reactive oxygen species. Therefore, the concentration of reactive oxygen species in tumor cells will increase with the intervention of a ketogenic diet [28]. Tumor cells are very vulnerable to reactive oxygen species and can be damaged [29].

The binding of insulin or free insulin-like growth factor IGF-1 to specific tyrosine kinase receptors can activate the insulin/IGF-1-PI3K-Akt-mTOR signaling pathway and enhance glycolysis and glutamine breakdown, thereby promoting tumor cell proliferation [27]. Meanwhile, activation of this signaling pathway can reduce ketogenesis by inhibiting intracellular peroxidase proliferators and activating receptor α (PPARα) [30]. However, a ketogenic diet that restricts carbohydrates and energy can counteract this effect by reducing ATP/AMP levels and activating the LKB1–AMPK–PPARα signaling pathway. On the one hand, AMPK inhibits mTORC1, and on the other hand, it collaborates with PPARα to reduce the expression of key enzymes of glycolipid and glutamine metabolism and inhibit glycolytic, thus specifically killing tumor cells [31,32]. The ketogenic diet can also suppress the mTOR signaling pathway, reducing inflammation and significantly reducing tumor growth [33].

This review used six randomized controlled trials, after rigorous observation and extraction from five different databases, for determining the effectiveness of LCKDs as an adjuvant treatment for cancer. The findings of this review were obtained through careful studying of each paper and their results by comparing the lipid profiles, tumor markers, ketosis, level of satisfaction, and the presence of adverse events. After a meticulous examination of the acquired results, it was found that most evidence did not have any statistical significance and therefore the effects of LCKDs as an adjuvant therapy on cancer management was inconclusive.

Only three other profiles were found to have statistical significance. These include the tumor marker PSA [25], the achievement of ketosis [14,23], and the level of satisfaction [14]. When comparing the tumor marker PSA between subgroups (non-KD and KD groups), those who were in the non-KD group had a better effect on their diet than the KD group. The level of satisfaction was also higher in the non-KD group. While studying the level of ketosis achieved between the subgroups, the KD group had a statistically higher occurrence of ketosis than the non-KD group. Since each paper used different parameters to measure the effects of the ketogenic diet as antitumor therapy, it was difficult to properly compare each parameter perfectly with all the RCT trials discussed. For instance, the total cholesterol was only used by three out of the six papers discussed because only those three papers had used total cholesterol to measure out the effects of a ketogenic diet compared to non-ketogenic diet.

The results showed that all the lipid profile contents including TC, HDL, LDL, TG, BW, FBG, and insulin had no proven statistical significance and, therefore, showing no difference in effects between the KD group and non-KD group. However, since all the RCTs discussed did not include all of the abovementioned lipid profile contents, it cannot be exclusively proven whether KD was effective or ineffective in improving lipid profile. Specific tumor markers were also presented, including CEA, CA 19-9, and PSA, which were also presented in the results; however, each of these tumor markers was only seen in or explained in one RCT, therefore not allowing further analysis. For instance, CEA and CA 19-9 were both described only in one RCT [13] which talked about pancreatobiliary cancer, while PSA was shown in another RCT [25] for prostate cancer and with no other comparison. Although PSA showed a better effect in the non-KD group, it cannot be proven to be effective because none of the other RCTs had measured PSA as a parameter and therefore should be inconclusive.

The level of satisfaction was also only measured in one out of the six RCTs [14] discussed. Even though the results showed that there was statistical significance in favor of the non-KD group, this result cannot be perfectly proven because there were no other papers to compare and prove the significance. The level of ketosis was an important indicator of whether the participants of the KD group were strictly consuming the ketogenic diet. Only two studies [14,23] were able to measure the level of ketosis and it was found to be statistically significant towards the KD group. Both RCTs measured the serum ketone bodies to evaluate the level of ketosis achieved by both the KD group and the non-KD group. Although the level of ketosis was a great indicator of strict ketogenic diet consumption, there were no definite indicators for non-KD groups and was therefore reliant on participants’ records of diet consumption [23].

The adverse effects of the ketogenic diet were only reported in three RCTs [14,23,25] but only two of the RCTs [14,23] were able to report the number of adverse events which occurred in the participants. When the reports were compared among these two RCTs, it was found to have no statistical significance between the two interventions. However, because the heterogeneity was very significant (I^2^ = 82%), this result may not necessarily be true. Since both RCTs had participants diagnosed with cancer, they have undergone treatment including chemotherapy and/or surgery. These therapies may have caused adverse effects during the consumption of KD or non-KD diets. There is no definite proof of whether these adverse effects observed during the interventions were specifically caused by the diets itself.

However, none of the mentioned results gave a good conclusion as to whether it was helpful as adjuvant antitumor therapy. Many factors have affected the results including different parameters measured within each discussed RCT. Therefore, it is difficult to obtain an accurate conclusion to dictate the ketogenic diet as adjuvant therapy for cancer.

There are some limitations of this review. Since all the papers were mostly heterogeneous, the results may not lead to the best conclusion. The profiles compared between all the included papers all had different types of cancers and treatment and, thus, it was difficult to compare and provide a conclusive result as the different cancer pathogenesis and pathophysiology may cause different responses to LCKDs. In the future, ketogenic diets must be studied in a setting with a less heterogeneous malignancy conditions, such as exploring similar types of cancer and when using a similar type of treatment modalities. All the papers discussed in this review had small populations for comparison. This may also not lead to the most accurate results. Therefore, when researching the fields of ketogenic diet and cancer, a bigger sample size must be conducted. It was difficult to compare all the papers in the same sequence because each measured a different type of parameter and, consequently, did not have any statistically significant results. Hence, more trials are necessary in the future, without the above limitations as much as possible, to collect congruent information for further elucidating the impact of LCKDs as an adjuvant in cancer management to arrive at a more informative conclusion.

## 5. Conclusions

The pooled results from the studies show inadequate evidence to support the beneficial effects of LCKDs on antitumor therapy. At present, there are not enough studies on the mechanism of the ketogenic diet. More studies are needed to clarify ketogenic diets’ efficacy and safety. We remain concerned about the practical use of the KD in cancer patients. More detailed practical guidance on the ketogenic diet in cancer patients is needed, such as timing of the intervention, patient age, the severity of the disease, and nutritional status. These factors may affect the efficacy of KD, but many previous studies have not addressed this. Besides, clinician and patient preference have to be taken into account. It is necessary to carry out more trials that include a bigger population to precisely compare the KD group and the non-KD group. It must include the same or similar types of cancers affecting the same organ, such as the ovaries, uterus, prostate, pancreas, etc., to have a precise comparison group. It would be a better solution for future trials to have a specific set of data, including the lipid profile, tumor markers, level of ketosis, adverse effects, and/or level of satisfaction which would allow the comparisons between each trial to be more accurate. It would also allow us to observe whether the KD diet had an effect against cancer and against which type of cancers it is more effective.

## Figures and Tables

**Table 1:** PICOS criteria for inclusion and exclusion of studies.

Parameter	Inclusion Criteria
Population	Cancer patients
Intervention/exposures	Dietary intake of ketogenic diet; low-carbohydrate diet
Comparison	Any comparison
Outcomes	Effectiveness, weight change, glucose level, insulin, lipid profiles, adverse outcome
Type of study	Randomized controlled trials

**Table 2:** Search terms used to identify articles related to ketogenic diet or related to human cancer.

1. KD *	21. Tumo *
2. Ketogenic *	22. Carcinoma *
3. Keto *	23. Malignan *
4. Low carb *	24. Ongolog *
5. Low-carb *	25. Metastas *
6. High fat *	26. Lymphoma *
7. High-fat *	27. leukemia
8. medium chain tryglyceride *	28. Adenoma *
9. medium chain triglyceride *	29. Adenocarcinoma*
10. MCT *	30. Glioma *
11. Atkin *	31. Sarcoma *
12. 2 or 3 or 4 or 5 or 6 or 7 or 8 or 9 or 10 or 11	32. 19 or 20 or 21 or 22 or 23 or 24 or 25 or 26 or 27 or 28 or 29 or 30 or 31
13. Diet *	33. Randomized controlled trial *
14. Plan *	34. Controlled clinical trial *
15. Treat *	35. Random *
16. 13 or 14 or 15	36. RCT *
17. 12 and 16	37. 33 or 34 or 35 or 36
18. 1 or 17	38. 18 and 32 and 37
19. Neoplasm *	
20. Cancer *	

**Table 3:** General characteristics of included studies in meta-analysis.

First Author/Year	Study Design	Types of Cancer	Concurrent Treatment	Interventions	Inclusion Criteria	Exclusion Criteria	Outcomes
OK (2018)	Prospective RCT	Pancreatic cancer Duodenal cancer Common bile duct cancer Ampulla of Vater cancer Cholangiocarcinoma Neuroendocrine tumor	Operation	KD: 3–6% of carbohydrate and 1 g/kg of high-quality protein was provided daily. 70–80% of energy was given through fat to achieve a ketogenic ratio of 1.05–1.75:1 (fat: carbohydrate + protein).	- Age ≥ 19 years old - Pancreatobiliary cancer who underwent pancreaticoduodenectomy or distal pancreatectomy	- Pregnant women - Illiterate patients - Severe diabetic complications - Hyperlipidemia with cardiovascular complications - Renal insufficiency with GFR < 90%	Meal compliance, energy and protein intake rates Meal satisfaction score Meal intake-related problems PG-SGA score Biochemical indices Body composition Urine ketone detection
Cohen (2018)	RCT	Ovarian/endometrial cancer	Chemotherapy	KD: ~5% pf energy from carbohydrate (≤20 g/d), 25% energy from protein (≤100 g/d) and 70% energy from fat (≥125 g/d)	- Age ≥ 19 years old - BMI ≥ 18.5 kg/m^2^ - No pre-existing medical conditions affecting body weight (other than cancer and associated treatment) - Not be currently attempting diet modification or weight loss/gain - No medical history contraindicating enrollment	Not mentioned	Dietary adherence Body composition Metabolic effects
Cohen (2020)	RCT	Ovarian/endometrial cancer	Chemotherapy	KD: ~5% pf energy from carbohydrate (≤20 g/d), 25% energy from protein (≤100 g/d) and 70% energy from fat (≥125 g/d)	- Age ≥ 19 years old - BMI ≥ 18.5 kg/m^2^ - No pre-existing medical conditions affecting body weight (other than cancer and associated treatment) - Not be currently attempting diet modification	Serious cardiovascular disease and events.	Serum lipids Serum ketone levels Adverse events Dietary intake Adherence
Khodabakhshi (2019)	RCT	Breast cancer	Chemotherapy	KD: MCT-based KD containing 6% calorie from CHO, 19% protein, 20% MCT, 55% fat	- Age 18–70 years old - Proven malignant biopsy - Undergoing chemotherapy for ≥3 months	- Significant cardiac, renal or neurologic comorbidities - Active state of malnutrition - Diabetes - Pregnancy - Karnofsky index <70	Metabolic profile Body composition Biochemical parameters Lipid profile Survival rate
Kang (2019)	Prospective RC	Pancreatic cancer Duodenal cancer Common bile duct cancer Ampulla of Vater cancer Neuroendocrine tumor	Operation	LCKD: Energy content: 1500 kcal/d, provided 4% from carbohydrate, 16% from protein and 80% from fat. Ketogenic ratio of 1.75:1 (F: C + P *w*/*w*).	- Age ≥ 19 years old - Pancreatobiliary cancer who underwent pancreaticoduodenectomy or distal pancreatectomy	- Pregnant women - Illiterate patients - Foreigner - Severe diabetic complications - Hyperlipidemia with cardiovascular complications - Renal insufficiency with GFR < 90%	Nutritional intake Blood biochemistry Non-targeted metabolomics analysis Lipid profile
Freedland (2019)	Multi-center phase II RCT	Prostate cancer	ADT	LCD/walking arm: carbohydrate intake ≤ 20 g/d and walk ≥ 30 min/day for ≥5 days/week. List of LC foods to choose from and list of moderate/high carbohydrate foods to limit. Sample menus and recipes were given. Coaching by dietitian in person or by phone weekly for months 0–3 and biweekly for months 4–6.	- Men initiating ADT for PCa with an anticipated duration of ≥6 months - BMI ≥ 24 kg/m^2^ - Phone access for calls	- Symptomatic metastatic disease - Medication controlled diabetes - Medications that interfere with insulin - Already having LCD - Vegetarian/vegan - Hemoglobin A1c >7%	Insulin resistance Weight, body composition, lipids, and prostate-specific antigen (PSA)

Abbreviations: ADT, androgen deprivation therapy; BMI, body mass index; GFR, glomerular filtration rate; KD, ketogenic diet; LC, low carbohydrate; LCD, low-carbohydrate diet; PCa, prostate cancer; Post-op, post-operation; RCT, randomized controlled trial.

**Table 4:** Baseline characteristics of patient comparing intervention group and control group.

First Author (Year)	Intervention	Number of Enrollment	Number of Completion	Mean (SD) Age at Baseline (Year)	BMI Mean (SD) In kg/m^2^ at Baseline	Duration (Mean Week)	Completion Rate on Diets at the End of Trial (%)
OK (2018)	KD GD	20 10	10 9	57.8 (7.3) 66.3 (9.8)	24.0 (2.2) 22.2 (2.6)	4	50.0 90.0
Cohen (2018)	KD ACS	37 36	25 20	61.5 (8.5) 58.6 (11.7)	30.7 (8.0) 33.0 (10.7)	12	55.6 67.6
Cohen (2020)	KD ACS	37 36	25 20	61.5 (8.5) 58.6 (11.7)	30.7 (8.0) 33.0 (10.7)	12	55.6 67.6
Khodabakhshi (2019)	KD SD	40 37	30 30	44.8 (8.4) 45.2 (15.0)	28.47 (4.1) 28.44 (5.8)	12	75.0 81.1
Kang (2019)	LCKD GD	20 10	9 9	58.3 (7.6) 66.3 (9.8)	24.0 (2.2) 22.2 (2.7)	4	45.0 90.0
Freedland (2019)	LCD GD	20 22	11 18	67.8 (12.7) 63.8 (11.3)	31.9 (9.5) 29.4 (4.0)	24	55.0 81.8

Abbreviations: ACS, American Cancer Society diet; BMI, body mass index; GD, general diet; KD, ketogenic diet; LCD, low-carbohydrate diet; LCKD, low-carbohydrate ketogenic diet; MCT, medium chain triglycerides; SD, standard diet.

## Data Availability

All data generated or analyzed during this study are included in this published article.

## Supplementary Materials

The following are available online at https://www.mdpi.com/article/10.3390/nu13051388/s1, Table S1: PRISMA 2009 checklist; Table S2: Detailed judgement for the risk of bias assessment.
