# Supplementary material for: Efficacy of Low-Carbohydrate Ketogenic Diet as an Adjuvant Cancer Therapy: A Systematic Review and Meta-Analysis of Randomized Controlled Trials
Source: Nutrients. 2021 Apr 21;13(5):1388. doi: 10.3390/nu13051388 (PMC8142992; doi:10.3390/nu13051388)
Supplement: Supplementary file 1 [file nutrients-13-01388-s001.zip › Supplementary File/Table S2-Detailed Judgement for Risk of Bias assessment.pdf]

Table S2: detailed judgement for risk of bias assessments.

Author	**Bias**	**Author's judgement**	**Support for judgement**
Cohen 2018	Random sequence generation (selection bias)	Low risk	Quote: "Participants were randomly assigned, through the use of a computer- generated blocked randomization scheme (created by KRF), to either the ACS or the KD group."
	Allocation concealment (Selection bias)	Low risk	Quote: "Participants were randomly assigned, through the use of a computer- generated blocked randomization scheme (created by KRF), to either the ACS or the KD group."
	Blinding of participants and researchers (performance bias)	High risk	Quote: "Because this was a diet intervention study, it was not possible for participants or study personnel to be blinded to group assignment."
	Blinding of outcome assessment (detection bias)	Unclear risk	Comment: Study did not address this outcome. Does not have information of whether this non-blinding method influences the outcome or not.
	Incomplete outcome data (attrition bias)	Low risk	Comment: 12 drop-out and the reasons for drop-out were disclosed and unlikely to affect final results. "those who withdrew did not differ from participants who completed the trial on BMI or fat mass at baseline (…)"
	Selective reporting (reporting bias)	Low risk	Quote: "This study was conducted as a randomized clinical trial (NCT03171506) with parallel arm design."<br>Comment: The study protocol was available and all of the study's pre-specified outcomes, that are of interest, have been reported.

	Other bias	High risk	Quote: "...heterogeneous nature of the sample...[and]... did not provide the food for participants, which detracts from our ability to better control dietary intake and adherence but enhances the generalizability of our results."

Author	Bias	Author's judgement	Support for judgement
Cohen 2020	Random sequence generation (selection bias)	Low risk	Quote: "Using a computer-generated blocked randomisation scheme, subjects were assigned to either the American Cancer Society diet (ACS) or the KD."
	Allocation concealment (Selection bias)	Low risk	Quote: "Using a computer-generated blocked randomisation scheme, subjects were assigned to either the American Cancer Society diet (ACS) or the KD."
	Blinding of participants and researchers (performance bias)	High risk	Quote: "Because this was a diet intervention study, it was not possible for participants or study personnel to be blinded to group assignment."
	Blinding of outcome assessment (detection bias)	Unclear risk	Comment: Insufficient information to permit judgement.
	Incomplete outcome data (attrition bias)	Low risk	Comment: No missing outcome data
	Selective reporting (reporting bias)	Low risk	Comment: Study protocol not clear but it included all expected outcomes, including those that were pre-specified.

	Other bias	High risk	Quote: "...sample was relatively heterogeneous in terms of cancer stage and treatment history. A small proportion of the study sample also received concurrent chemotherapy, which presents a potential obstacle to dietary adherence,... assessment of adherence relied upon diet records from subjects, which may be subject to under-reporting and errors... [, and] this study is limited by its small sample size..."

Author	**Bias**	**Author's judgement**	**Support for judgement**
Freedland 2019	Random sequence generation (selection bias)	Low risk	Quote: "Randomization was conducted by permutated block design, stratified by center, and whether the participant received concurrent radiation for PCa."
	Allocation concealment (Selection bias)	Unclear risk	Comment: Insufficient information to permit judgement.
	Blinding of participants and researchers (performance bias)	High risk	Quote: "The leading investigator (SJF) was blinded to the randomization and not involved in data collection."<br>Quote: "Some patients refused to participate in the study because they consider dietary intervention as an additional burden despite the potential benefit. On the other hand, some refused to participate because of the possibility of being randomized to the control group."
	Blinding of outcome assessment (detection bias)	Unclear risk	Comment: Insufficient information to permit judgement.
	Incomplete outcome data (attrition bias)	Low risk	Comment: No missing outcome data.

	Selective reporting (reporting bias)	Low risk	Quote: "The primary outcome was percent change in HOMA at 6 months between arms, which was tested using the rank-sum test[.] Secondary outcomes included changes in anthropometric and various health measures from baseline to 3 and 6 months, which were compared using a rank-sum test."
	Other bias	High risk	Quote: "The underpowered sample size likely contributed to our overall negative findings."

**Author**	**Bias**	**Author's judgement**	**Support for judgement**
Kang 2019	Random sequence generation (selection bias)	Unclear risk	Quote: "After screening 47 patients for eligibility, 30 patients voluntarily enrolled and were randomly assigned to receive GD or LCKD."<br>Comment: Insufficient information provided about sequence generation process to permit judgement
	Allocation concealment (Selection bias)	Unclear risk	Comment: Insufficient information provided about sequence generation process to permit judgement.
	Blinding of participants and researchers (performance bias)	High risk	Comment: Dietary intervention not able to blind
	Blinding of outcome assessment (detection bias)	Unclear risk	Comment: Insufficient information to permit judgement.
	Incomplete outcome data (attrition bias)	High risk	Comment: 12 drop-out, the reasons were disclosed. Different loss of participants in intervention and comparison group was significant. Losses were likely to affect the final result.

	Selective reporting (reporting bias)	Low risk	Comment: Study protocol available and all of study's pre-specified outcomes, that are of interest, have been reported
	Other bias	Unclear risk	Comment: Insufficient information to assess whether an important risk of bias exists

Author	**Bias**	**Author's judgement**	**Support for judgement**
Khodabakhshi 2019	Random sequence generation (selection bias)	Low risk	Quote: "...were randomly selected and randomized using block balanced randomization in a 1:1 ratio into the intervention (n=40) and control groups (n=40)."
	Allocation concealment (Selection bias)	Low risk	Quote: "This protocol was computer-generated by a statistician who was not working with the patients."
	Blinding of participants and researchers (performance bias)	Unclear risk	Quote: "In the control group, placebo was a regular diet containing 55% CHO, 15% protein, and 30% fat.<br>Comment: Diet is impossible to keep patients blind but since the control group had placebo diet, participants might not have known which group they were placed in and if it had an effect on the paper.
	Blinding of outcome assessment (detection bias)	Unclear risk	Comment: Insufficient information to permit judgement.
	Incomplete outcome data (attrition bias)	Low risk	Comment: Reasons for those who withdrew were provided

	Selective reporting (reporting bias)	Low risk	Comment: Study protocol not available but published reports include all expected outcomes.
	Other bias	Unclear risk	Quote: "...the sample size is low, hence larger trials are needed." Comment: Insuffficient information to assess the level of bias.

**Author**	**Bias**	**Author's judgement**	**Support for judgement**
OK 2018	Random sequence generation (selection bias)	High risk	Quote: "…strict randomization was not possible because participants were patients who needed immediate cancer surgeries."
	Allocation concealment (Selection bias)	Unclear risk	Comment: Not reported what type of method used to allocate the participants.
	Blinding of participants and researchers (performance bias)	High risk	Comment: Since dietary intervention, it is not possible to blind participants.
	Blinding of outcome assessment (detection bias)	Low risk	Quote: "Anthropometric measurements, nutritional evaluation, and body composition analysis were performed before surgery (preOP), before discharge (DC), and at the first outpatient follow-up day (OPD) for all patients and general information (e.g., sex, age, diagnosis, type of surgery), blood and urine test results, and other relevant data were collected from electronic medical records".
	Incomplete outcome data (attrition bias)	High risk	Comment: 11 drop-out, the reasons were disclosed. Different loss of participants in intervention and comparison group was significant. Losses were likely to affect the final result.

	Selective reporting (reporting bias)	Low risk	Comment: Study protocol available and all of study's pre-specified outcomes, that are of interest, have been reported
	Other bias	Unclear risk	Quote: "…a larger number of participants are needed to assess the effects of KD as an adjuvant anti-cancer therapy."
